# Butterfly glioblastoma: trends in therapeutic modalities, extent of resection and survival in the temozolomide era. a SEER-based study

**DOI:** 10.1007/s10143-025-03558-2

**Published:** 2025-05-08

**Authors:** Yosef Laviv, Ekkehard M. Kasper

**Affiliations:** 1 Rabin Medical Center, Department of Neurosurgery, Tel-Aviv University, Beilinson Hospital, Zeev Jabutinsky Rd 39, Petah Tikva, Petah Tikva, 49100 Israel; 2 Boston Medical Center Healthcare Systems, Department of Neurosurgery Boston, Boston University, Boston, MA USA; 3 Faculty of Health Sciences, McMaster University, Hamilton, ON Canada

**Keywords:** Butterfly glioblastoma, Corpus callosum, Chemotherapy, Gross total resection

## Abstract

Butterfly glioblastoma (bGBM) is a rare type of GBM, thought to have extremely poor outcome and is generally considered “inoperable”. The aim of this study was to investigate survival outcomes in bGBM over a long period, and to look for potential benefits of gross total resection (GTR). The data of 521 bGBM patients diagnosed between 2000 and 2021 from the Surveillance, Epidemiology, and End Results (SEER) database were obtained. The cohort was divided into 3 subgroups based on the year of diagnosis: pre-temozolomide era (pre-TMZ; 2000–2006), early TMZ era (2007–2016) and late TMZ era (2017–2021). chemotherapy was significantly more common in the TMZ group (51.9% vs. 36%, *p* = 0.007). The TMZ group was associated with nearly significantly improved OS (HR = 0.795, *p* = 0.067). On multivariate analysis, only radiation therapy was associated with improved survival in the pre-TMZ group (HR = 3.029, *p* = 0.001). However, in the TMZ group, 4 variables were associated with improved survival: chemotherapy (HR = 1.523, *p* = 0.049), radiation therapy (HR = 1.676, *p* = 0.006), surgery (HR = 1.402, *p* = 0.004) and age (HR = 1.031, *p* < 0.001). Both subtotal resection and GTR were significantly associated with favorable prognosis when compared to no surgery (HR = 0.607, *p* < 0.001 and HR = 0.467, *p* < 0.001; respectively). The rate of GTR was significantly higher in the late TMZ group (19.2% vs. 10.2%, *p* = 0.0013). GTR was significantly associated with improved OS in the late TMZ subgroup (HR = 1.846, *p* = 0.028). bGBM cases in the TMZ era are associated with increased rate of adjuvant chemotherapy as well as with improved OS, in comparison to pre-TMZ cases. The rate of GTR cases has significantly increased in recent years, and is associated with significantly increased rate of post operative adjuvant therapy. GTR patients who have received any kind of adjuvant therapy had significantly better OS when compared to non-GTR patients. bGBM should be treated like other operable GBM cases, using the appropriate, advanced surgical techniques.

Clinical trial number: Not applicable.

**Supplementary Information:**

The online version contains supplementary material available at 10.1007/s10143-025-03558-2.

## Introduction

Glioblastoma (GBM) is the most common malignant primary brain tumor and carries a dismal prognosis. Median survival is 15–20 months even with intensive modern treatment that includes maximal safe resection, chemotherapy, and radiation [1].

In 2005, Stupp et al. have published their landmark paper on the preferred adjuvant protocol for GBM. Following this study, post operative treatment with radiation and temozolomide (TMZ) has become the standard protocol worldwide.

Butterfly glioblastoma (bGBM) is a rare manifestation of GBM, characterized by a butterfly pattern of lesional extent on imaging, due to bihemispheric involvement and invasion of the corpus callosum (CC) [2]. The prevalence of this radiologic subtype of GBM is only 2-6% of all GBM cases [2–6]. The frequencies of the various sites involved are as follows: the genu/rostrum of the CC (frontal, 41.9%), the body of CC (fronto– parietal, 22.6%) and the splenium (parietal, 35.5%) [4]. CC involvement on pre-operative imaging is recognized as 1 out of 5 radiographic features mostly associated with poor surgical outcome [7]. In a large national cancer database study, median OS was significantly shorter in bGBM cases compared to most regular GBMs (7.1 months vs. 13.2 months, *p* < 0.001) [4]. Only 9% of patients with bGBM survive beyond 2-years [8].

Early attempts at surgical resection of bGBM caused devastating neurologic deficits, including abulia and akinetic mutism from cases involving the genu or agraphia without alexia from splenial bGBM [6]. In many institutions, attempting surgical resection of bGBM has only started in the last decade [3]. Nevertheless, increasing evidence from surgical series indicates that with current surgical and anesthesia tools, resection of bGBM can be performed safe and translates into a survival benefit [2, 3, 9]. In a study on 25 bGBM patients, 14.5% had gross-total resections (GTR) and 30.9% had partial resections. The 2-year survival rate after resection was 30% compared to 7% after biopsy (*p* = 0.047). The major benefit was achieved in the group with GTR, while partial resection failed to improve survival [9]. In 2020, evidence-based guidelines on the role of cytoreductive surgery in newly diagnosed GBM have stated that maximal resection was recommended over biopsy alone for both supratentorial cases in general (class II evidence level) as well as for bGBM cases (class III evidence level) [10].

Due to the rarity of bGBM and the currently prevailing opinion in the neurosurgical and neurooncological communities that major resections should not be attempted, we are facing a paucity of current literature on surgical resection and outcome of bGBM which is further limited by the fact that these reports are from small case series.

In order to obtain a wider view on possible trends in surgical outcomes and to assess prognosis, we set out to use a much larger cohort of bGBM cases from the Surveillance, Epidemiology, and End Results (SEER) database. Specifically, we have looked for changes in employed therapeutic modalities, reported extent of resection (EOR) and overall survival during three key periods: the pre-TMZ era (= pre-TMZ; years 2000–2006), early TMZ era (years 2007–2016) and late TMZ era (years 2017–2021).

## Materials and methods

Study data were obtained using the SEER*Stat software (version 8.4.4). The SEER database, managed by the National Cancer Institute, is a comprehensive and authoritative source of cancer data in the United States. These data are publicly accessible and available for free with an approved application. We obtained authorization from the National Cancer Institute to access the SEER dataset for research purposes only (reference number: SAR0090733- October 2024). Since the SEER database anonymizes patient information, the extraction of data for this study did not require informed consent, nor was ethical approval needed. Through the “Incidence-SEER Research Data, 17 Registries, Nov 2023 Sub (2000–2021),” patients diagnosed with GBM between the years 2000 and 2021 were identified. The International Classification of Diseases for Oncology (ICD-O-3) histologic codes (9440,9441) and the Seer Brain and CNS recode (1.1.2) were used for GBM diagnosis (*n* = 59,010). In order to keep in line with the most recent (2021) World Health Organization (WHO) classification of tumors of the central nervous system [11], the diagnosis of Glioblastoma, IDH-mutant (ICD-0-3 code 9445) was excluded as it is no longer considered as GBM. Primary sites within central nervous system were identified by site-specific codes (C71.1–C71.8). Only cases coded as C71.1 (frontal) and C71.3 (parietal) were included in this analysis. Finally, under “Site and Morphology, laterality”, only cases coded as “Bilateral, single primary” or “Paired site: midline tumor” were included, yielding a final cohort of bGBM cases (*n* = 521).

### Variables

Data of all eligible patients were extracted from the SEER database records as follows: demographic (year of diagnosis, age at diagnosis, gender, race and marital status); clinical (radiation therapy performed vs. no/unknown, chemotherapy performed vs. no/unknown, time from diagnosis to treatment, cause of death and 6months, 12months and overall survival ); radiological (tumor’s size at diagnosis, anatomical location frontal vs. parietal) and surgical (surgery performed or recommended vs. no or unknown, extent of resection). The cohort was initially divided into 2 subgroups based on year of diagnosis: pre-TMZ era (2000–2006) and TMZ era (2007–2021).

For extent of resection - based analyses, the TMZTMZ group was further divided into early TMZ era (2007–2016) and late TMZ era (2017–2021).

Overall survival (OS) was defined as the time from the date of diagnosis to death (non-censored) or last follow-up (censored). Extreme cases with “0” month or > 60months OS were excluded from survival analyses.

### Statistical analysis

Descriptive statistics were employed to analyze the attributes of the study population. Each variable was presented by the most suitable central and dispersion measures: nominal variables were presented by number and percent (%), numerical variables were presented by mean ± standard deviation (SD). Normal distribution of numerical variables was assessed using histograms, Q-Q plots, Shapiro-Wilk test, and Kolmogorov-Smirnov test. First, we conducted univariate analysis to assess the clinical and sociodemographic characteristics of the study cohort stratified by year of diagnosis. For continuous variables we used Man-Whitney test suitable for non-normal distribution, and for nominal variables we used either Chi-square test or Fisher’s exact test. Furthermore, we examined the association between different variables and all-cause mortality using Kaplan-Meier curves and Log-rank test. Finally, we used univariate and multivariable Cox regression to assess Hazard ratios (HR) for OS of select variables, and stratified this analysis by the different time periods under consideration (i.e. pre-TMZ, early TMZ and late TMZ). For secondary analysis, similar steps were performed for the entire cohort, stratified by extent of resection (GTR vs. non-GTR).

All analyses were conducted using SPSS Statistics (IBM, Armonk, NY, USA; version 28) and R software. A two-sided test significance level of 0.05 was used throughout the entire study.

## Results

A total of 521 cases were eligible for statistical analysis. Mean age at diagnosis was 62.9 ± 13.0 years, with a small male predominance (M: F = 1.25). The subgroup sizes were *n* = 376 for frontal and *n* = 47 for parietal bGBM cases (72.1% and 28.9% of the entire cohort, respectively).

For primary analysis, the cases from the years 2000–2006 (= pre-TMZ era, *n* = 89) were compared to cases from the years 2007–2021 (= TMZ era, *n* = 432) (Table 1). As expected, chemotherapy was significantly more common in the aggregate TMZ subgroup (51.9% vs. 36%, *p* = 0.007). There were also some minor, yet significant, demographic changes between the two subgroups, as the reported cases from the pre-TMZ showed a female predominance and a higher incidence of younger patients (< 50 years of age). Interestingly, no other significant differences were found between the two groups.

For secondary comparison, the TMZ group was further divided into early TMZ (2007–2016, *n* = 271) and late TMZ (2017–2021, *n* = 161) (Table 1). Here, the chemotherapy utilization rate was nearly identical among the two subgroups (~ 50%, *p* = 0.842). The rate of performed surgery was higher in the late– vs. early TMZ groups, but only reached near significance (52.1% vs. 43.5%, *p* = 0.073). On the other hand, the rate of reported GTR was significantly higher in the late TMZ compared to the early TMZ subgroup (19.2% and 10.3%, respectively; *p* = 0.013). In addition, the rate of any adjuvant therapy was significantly higher in the late TMZ group (36.6% vs. 26.9, *p* = 0.040). Finally, the incidence of GBM as a cause of death was significantly higher in the early TMZ group (93.0% vs. 77.0%, *p* < 0.001).

**Table 1:** Butterfly glioblastoma– differences in demographic, radiologic, surgical and clinical parameters, dependent on different periods at time of diagnosis

	Year of diagnosis							
	pre– Temozolomide era vs. Temozolomide l era				Early Temozolomide era vs. Late Temozolomide protocol era			
Variables	Total (*n* = 521)	Years 1999–2006 (*n* = 89)	Years 2007–2021 (*n* = 432)	*P* value*	Total (*n* = 432)	Years 2007–2016 (*n* = 271)	Years 2017–2021 (*n* = 161)	*P* value*
**Age at diagnosis**,** y (mean ± SD)**	62.99 ± 13.01	61.18 ± 12.49	63.36 ± 13.10	0.140	63.36 ± 13.10	63.17 ± 13.38	63.68 ± 12.65	0.692
**Gender ratio (M: F)**	1.25 (290:233)	0.81 (40:49)	1.37 (250:182)	**0.027**	1.37 (250:182)	1.46 (161:110)	1.23 (89:72)	0.421
**Age**,** n (%)** < 50 50–59 60–69 70–79 ≥ 80	72 (13.8) 127 (24.4) 161 (30.9) 106 (20.3) 55 (10.6)	19 (21.3) 21 (23.6) 22 (24.7) 21 (23.6) 6 (6.7)	53 (12.3) 106 (24.5) 139 (32.2) 85 (19.7) 49 (11.3)	**0.028** 0.893 0.207 0.389 0.255	53 (12.3) 106 (24.5) 139 (32.2) 85 (19.7) 49 (11.3)	38 (14.0) 63 (23.2) 85 (31.4) 54 (19.9) 31 (11.4)	15 (9.3) 43 (26.7) 54 (33.5) 31 (19.3) 18 (11.2)	0.173 0.420 0.670 0.901 1.000
**Race**,** n (%)** White Other	471 (90.4) 50 (9.6)	82 (92.1) 7 (7.9)	389 (90.0) 43 (10.0)	0.693	389 (90.0) 43 (10.0)	241 (88.9) 30 (11.1)	148 (91.9) 13 (8.1)	0.406
**Marital status**,** n (%)** Married Divorced Single (never married) Widowed	333 (65.7) 49 (9.7) 82 (16.2) 43 (8.5)	58 (65.9) 11 (12.5) 11 (12.5) 8 (9.1)	275 (65.6) 38 (9.1) 71 (16.9) 35 (8.4)	0.610	275 (65.6) 38 (9.1) 71 (16.9) 35 (8.4)	174 (65.4) 19 (7.1) 49 (18.4) 24 (9.0)	101 (66.0) 19 (12.4) 22 (14.4) 11 (7.2)	0.230
**Tumor’s size at diagnosis**,** mm (mean ± SD)**	52.40 ± 16.83	53.98 ± 17.02	52.12 ± 16.81	0.423	52.12 ± 16.81	52.86 ± 16.86	50.88 ± 16.70	0.280
**Surgery** No\Unknown Performed or recommended	265 (50.9) 255 (49.1)	45 (51.1) 43 (48.9)	220 (50.9) 212 (49.1)	1.000	220 (50.9) 212 (49.1)	147 (54.2) 124 (45.8)	73 (45.3) 88 (54.7)	*0.091*
**Surgery**,** n (%)** **GTR** **non-GTR** **GTR** **No surg. or non-GTR**	242 (45.9) 69 (28.5) 173 (71.5) 69 (13.6) 438 (86.4)	40 (46.9) 10 (25) 30 (75) 10 (11.8) 75 (88.2)	202 (46.7) 59 (29.2) 143 (70.8) 59 (14.0) 363 (86.0)	0.983 0.846 0.729	202 (46.7) 59 (29.2) 143 (70.8) 59 (13.6) 373 (86.4)	118 (43.5) 28 (23.7) 90 (76.3) 28 (10.3) 243 (89.7)	84 (52.1) 31 (36.9) 53 (63.1) 31 (19.2) 130 (80.8)	*0.073* **0.050** **0.013**
**Radiation therapy**,** n (%)** No\Unknown Performed	356 226 (63.5) 130 (36.5)	63 42 (66.7) 21 (33.3)	293 184 (62.8) 109 (37.2)	0.666	293 184 (62.8) 109 (37.2)	187 110 (58.8) 77 (41.2)	106 74 (69.8) 32 (30.2)	*0.078*
**Combined therapy**,** n (%)** **Rad after surg** **Rad without surg** Any adjuvant therapy ** **No adjuvant therapy or no surg.**	295 165 (55.9) 130 (44.1) N/A N/A	47 26 (55.3) 21 (44.7) N/A N/A	248 139 (56.0) 109 (44.0) 132 (30.6) 300 (69.4)	0.925 N/A	248 139 (56.0) 109 (44.0) 132 (30.6) 300 (69.4)	161 84 (52.2) 77 (47.8) 73 (26.9) 189 (73.1)	87 55 (63.2) 32 (36.8) 59 (36.6) 102 (63.4)	0.108 **0.040**
**Chemotherapy**,** n (%)** No\Unknown Performed	521 265 (50.9) 256 (49.1)	89 57 (64.0) 32 (36.0)	432 208 (48.1) 224 (51.9)	**0.007**	432 208 (48.1) 224 (51.9)	271 129 (47.6) 142 (52.4)	161 79 (49.1) 82 (50.9)	0.842
**Time from diagnosis to treatment**,** d (mean ± SD)**	12.24 ± 22.59	9.65 ± 13.16	12.74 ± 23.92	0.330	12.74 ± 23.92	12.04 ± 15.06	13.90 ± 33.91	0.509
**Cause of death**,** n (%)** GBM Alive\Unknown \Other cause	459 (88.1) 62 (11.9)	83 (93.3) 6 (6.7)	376 (87.0) 56 (13.0)	0.108	376 (87.0) 56 (13.0)	252 (93.0) 19 (7.0)	124 (77.0) 37 (23.0)	**< 0.001**
**Anatomical location**,** n (%)** Frontal Parietal	464 (89.1) 57 (10.9)	78 (87.6) 11 (12.4)	386 (89.4) 46 (10.6)	0.709	386 (89.4) 46 (10.6)	237 (87.5) 34 (12.5)	149 (92.5) 12 (7.5)	0.108

d = days; F = female; GBM = glioblastoma; GTR = gross total resection; M = male; mm = millimeter; n = number; NA = not available; Rad = radiotherapy; SD = standard deviation; surg = surgery; y = years

* Note: Boldface type indicates *p* < 0.05; Italic type indicated near significance

** Note: the adjuvant data is only available for the years 2007–2021 (there is no data for earlier years)

In a third analysis step, GTR (*n* = 69) and non-GTR (*n* = 453) groups were compared (Table 2). Mean age at diagnosis was significantly lower in the GTR group (55.9 ± 12.5 vs. 64.0 ± 12.8, *p* < 0.001) and the fraction of young patients (< 50 years of age) was significantly higher in the GTR group (30.4% vs. 11.3%, *p* < 0.001). The incidence of radiotherapy, chemotherapy or any combination of these treatments with surgery was significantly higher in the GTR group. Finally, the rate of GBM as a cause of death was significantly lower in the GTR group (71.0% vs. 90.0%, *p* < 0.001).

**Table 2:** Butterfly glioblastoma– hazard ratios for the different variables, dependent on the different periods at time of diagnosis: a Cox regression, univariate analysis

	Year of diagnosis							
	Total cohort		Pre Temozolomide era		Post Temozolomide era			
			1999–2006		2007–2016 (early)		2017–2021 (late)	
Variable	HR (95% CI)	P value*	HR (95% CI)	P value	HR (95% CI)	**P value**	HR (95% CI)	P value
**Sex**								
Male Female	Reference 1.363 (1.116–1.664)	**0.002**	Reference 1.467 (0.890–2.415)	0.133	Reference 1.555 (1.182–2.047)	**0.002**	Reference 1.080 (0.742–1.574)	0.687
**Age** < 50 50–59 60–69 70–79 ≥ 80	Reference 1.443 (1.025–2.030) 1.826 (1.317–2.530) 3.658 (2.536–5.277) 4.957 (3.156–7.786)	**< 0.001** **0.035** **< 0.001** **< 0.001** **< 0.001**	Reference 0.717 (0.348–1.475) 1.119 (0.563–2.225) 2.252 (1.068–4.751) 1.974 (0.644–6.050)	**0.046** 0.366 0.749 **0.033** 0.234	Reference 1.436 (0.905–2.280) 1.696 (1.095–2.626) 3.101 (1.899–5.064) 4.496 (2.490–8.119)	**< 0.001** 0.124 **0.018** **< 0.001** **< 0.001**	Reference 2.767 (1.184–6.467) 3.951 (1.726–9.043) 10.378 (4.168–24.840) 14.154 (4.970-40.311)	**< 0.001** **0.019** **0.001** **< 0.001** **< 0.001**
**Marital status** Single (never married) Married Divorced Widowed	Reference 1.189 (0.897–1.576) 1.292 (0.843–1.982) 2.786 (1.777–4.367)	**< 0.001** 0.228 0.240 **< 0.001**	Reference 1.110 (0.556–2.218) 0.854 (0.322–2.268) 2.656 (0.869–8.117)	0.265 0.767 0.752 *0.087*	Reference 0.970 (0.681–1.382) 1.152 (0.634–2.092) 2.120 (1.189–3.779)	**0.027** 0.866 0.642 **0.011**	Reference 1.836 (0.954–3.532) 2.014 (0.847–4.786) 5.837 (2.207–15.437)	**0.005** *0.069* 0.113 **< 0.001**
**Tumor’s size at diagnosis**,** mm**	1.002 (0.995–1.009)	0.557	0.998 (0.981–1.015)	0.832	1.002 (0.993–1.012)	0.607	1.000 (0.987–1.013)	0.976
**Surgery**								
Performed Not Performed **GTR** **non-GTR** **GTR** **No surg. or non-GTR**	Reference 1.764 (1.445–2.154) Reference 1.316 (0.953–1.818) Reference 1.687 (1.254–2.269)	**< 0.001** *0.095* **< 0.001**	Reference 1.732 (1.048–2.864) Reference 1.706 (0.786–3.703) Reference 2.021 (1.002–4.074)	**0.032** 0.177 **0.049**	Reference 1.573 (1.202–2.057) Reference 0.947 (0.602–1.490) Reference 1.222 (0.804–1.857)	**< 0.001** 0.814 0.348	Reference 2.421 (1.653–3.547) Reference 1.719 (0.954–3.095) Reference 2.393 (1.400-4.091)	**< 0.001** *0.071* **0.001**
**Radiation therapy**								
Performed No\Unknown	Reference 2.641 (2.126–3.280)	**< 0.001**	Reference 3.801 (2.127–6.792)	**< 0.001**	Reference 2.351 (1.746–3.166)	**< 0.001**	Reference 3.089 (2.064–4.623)	**< 0.001**
**Combined therapy**								
Rad after surg Rad without surg **Any adjuvant therapy** **No adjuvant therapy or no surg.**	Reference 1.741 (1.363–2.224) Reference 2.313 (1.825–2.930)	**< 0.001** **< 0.001**	Reference 1.988 (1.068-3.700) NA NA	**0.030**	Reference 1.420 (1.030–1.959) Reference 1.979 (1.471–2.662)	**0.032** **< 0.001**	Reference 2.750 (1.674–4.519) Reference 3.144 (2.107–4.689)	**< 0.001** **< 0.001**
**Chemotherapy**								
Performed No\Unknown	Reference 2.522 (2.049–3.103)	**< 0.001**	Reference 2.606 (1.544–4.398)	**< 0.001**	Reference 2.255 (1.701–2.989)	**< 0.001**	Reference 3.129 (2.105–4.650)	**< 0.001**
**Time from diagnosis to treatment**,** d**	0.999 (0.996–1.003)	0.785	0.995 (0.975–1.015)	0.610	1.001 (0.992–1.010)	0.805	0.999 (0.995–1.004)	0.780
**Anatomical location**								
Frontal Parietal	Reference 1.275 (0.734–2.647)	0.369	Reference 1.348 (0.664–2.738)	0.409	Reference 1.163 (0.774–1.746)	0.468	Reference 1.440 (0.725–2.860)	0.298

d = days; GBM = glioblastoma; GTR = gross total resection; mm = millimeter; Rad = radiotherapy; surg = surgery; y = years

* Note: Boldface type indicates *p* < 0.05; Italic type indicated near significance

It should be noted that there were no significant differences between frontal vs. parietal bGBM cases in terms of surgical, radiological or chemotherapeutic features (supplementary data, Table 1s)

For survival analysis, patients with an OS of “0” or patients with extreme OS (> 60months) were censored from the general cohort in order to achieve homogeneity and to minimize shift of median OS from mean OS. Thus, the final number of cases for survival analyses was 423. Table 3 summarizes the univariate analyses for different variable in each of the distinct periods. In the TMZ subgroups, older age was significantly associated with greater HR. In addition, patients who identified “widowed” as marital status showed a significant association with greater HR. Having no surgery was associated with greater HR in all subgroups, and GTR was significantly associated with lower HR, except in the early TMZ. Radiotherapy, chemotherapy or any combination of these treatments with surgery were significantly associated with lower HR in all subgroups.

**Table 3:** Butterfly glioblastoma– differences in demographic, radiologic, surgical and clinical parameters, dependent on extent of resection

Variable	GTR (*n* = 69)	non-GTR (*n* = 453)	*p* value*
**Age at diagnosis**,** y (mean ± SD)**	55.91 ± 12.46	64.03 ± 12.76	**< 0.001**
**Gender ratio (M: F)**	1.22 (38:31)	1.26 (252:199)	0.897
**Age**, n (%)			
< 50 50–59 60–69 70–79 ≥ 80	21 (30.4) 21 (30.4) 17 (24.6) 9 (13.0) 1 (1.4)	51 (11.3) 106 (23.5) 144 (31.9) 97 (21.5) 53 (11.8)	**< 0.001** 0.228 0.264 0.146 **0.005**
**Race**,** n (%)** White Other	61 (88.4) 8 (11.6)	409 (90.7) 42 (9.3)	0.514
**Marital status**,** n (%)** Married Divorced Single (never married) Widowed	44 (66.7) 8 (12.1) 11 (16.7) 3 (4.5)	289 (65.7) 40 (9.1) 71 (16.1) 40 (9.1)	0.579
**Tumor’s size at diagnosis**,** mm (mean ± SD)**	49.86 ± 16.60	52.81 ± 16.86	0.213
**Radiation therapy**,** n (%)** No\Unknown Performed	20 (29.0) 49 (71.0)	205 (45.5) 246 (54.5)	**0.013**
**Combined therapy**,** n (%)** Rad after surg Rad without surg **Any adjuvant therapy** **No adjuvant therapy or no surg.**	49 (100) 0 (0.0) 37 (62.7) 22 (37.3)	116 (47.2) 130 (52.8) 95 (25.5) 278 (74.5)	**< 0.001** **< 0.001**
**Chemotherapy**,** n (%)** No\Unknown Performed	27 (39.1) 42 (60.9)	237 (52.5) 214 (47.5)	**0.039**
**Time from diagnosis to treatment**,** d (mean ± SD)**	12.57 ± 43.69	12.16 ± 13.90	0.893
**Cause of death**,** n (%)** GBM Alive\Unknown \Other cause	49 (71.0) 20 (29.0)	410 (90.9) 41 (9.1)	**< 0.001**
**Anatomical location**,** n (%)** Frontal Parietal	60 (87.0) 9 (13.0)	403 (89.4) 48 (10.6)	0.537

d = days; F = female; GBM = glioblastoma; GTR = gross total resection; M = male; mm = millimeter; n = number; Rad = radiotherapy; SD = standard deviation; surg = surgery; y = years

* Note: Boldface type indicates *p* < 0.05; Italic type indicated near significance

Two multivariate, Cox regression analyses were performed. Table 4 summarizes the results of multivariate analysis using the variables chemotherapy, radiotherapy, surgery and age in the pre-TMZ and TMZ groups. As shown, only radiotherapy was associated with improved survival in the pre-TMZ group (HR = 3.029, *p* = 0.001). However, in the TMZ group, all four variables were associated with improved survival: chemotherapy (HR = 1.523, *p* = 0.049), radiation therapy (HR = 1.676, *p* = 0.006), surgery (HR = 1.402, *p* = 0.004) and age (HR = 1.031, *p* < 0.001).

**Table 4:** Butterfly glioblastoma– hazard ratios for selected variables: pre-Stupp protocol era vs. Stupp protocol era. A Cox regression, multivariate analysis

	pre-Temozolomide era (2000–2006)		Temozolomide era (2007–2021)	
Variable	HR (95% CI)	*P* value*	HR (95% CI)	*P* value
Chemotherapy				
Performed No\Unknown	Reference 1.805 (0.953–3.420)	0.070	Reference 1.523 (1.011–2.047)	0.049
**Radiotherapy**				
Performed No\Unknown	Reference 3.029 (1.556–5.898)	**0.001**	Reference 1.676 (1.160–2.422)	**0.006**
**Surgery**				
Performed Not Performed	Reference 1.665 (0.977)	*0.061*	Reference 1.402 (1.111–1.770)	**0.004**
**Age**,** y**	1.015 (0.987–1.043)	0.297	1.031 (1.019–1.042)	**< 0.001**

* Note: Boldface type indicates *p* < 0.05; Italic type indicated near significance

Table 5 summarizes the results of the multivariate analysis using the variables chemotherapy, radiotherapy and surgery (GTR vs. non-GTR) in the early TMZ and late TMZ groups. As shown, chemotherapy (HR = 1.702, *p* = 0.006) and radiotherapy (HR = 1.702, *p* = 0.021) remained significant in the early TMZ group. On the other hand, non-GTR was significantly associated with greater HR only in the late TMZ group (HR = 1.846, *p* = 0.028).

**Table 5:** Butterfly glioblastoma– hazard ratios for selected variables: early Temozolomide era vs. late Temozolomide era. A Cox regression, multivariate analysis

	Early years (2007–2016)		Late years (2017–2021)	
Variable	HR (95% CI)	*P* value	HR (95% CI)	*P* value
Chemotherapy				
Performed No\Unknown	Reference 1.702 (1.161–2.495)	0.006	Reference 1.888 (0.959–3.716)	0.066
**Radiotherapy**				
Performed No\Unknown	Reference 1.702 (1.161–2.495)	**0.021**	Reference 1.622 (0.819–3.210)	0.165
**Surgery**				
GTR non GTR	Reference 1.226 (0.807–1.864)	0.340	Reference 1.846 (1.067–3.194)	**0.028**

* Note: Boldface type indicates *p* < 0.05; Italic type indicated near significance

**Fig. 1:**
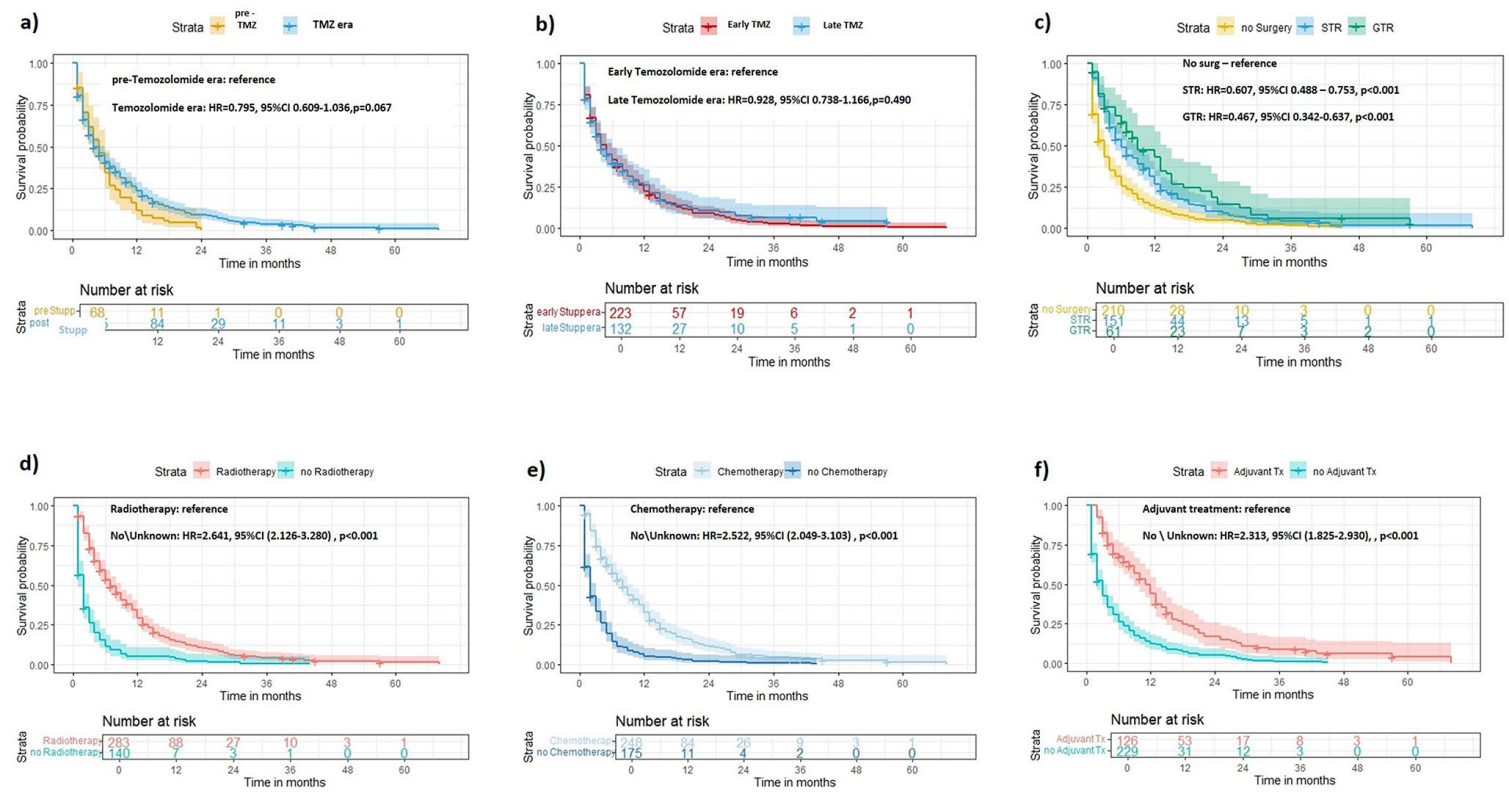
Kaplan-Meier survival curves, comparing different subgroups of bGBM cases. **a**) pre- Temozolomide era vs. Temozolomide era. **b**) early Temozolomide era vs. late Temozolomide era. **c**) No surgery vs. subtotal resection vs. gross total resection. **d**) Cases treated with radiotherapy vs. no or unknown radiotherapy. **e**) Cases treated with chemotherapy vs. no or unknown chemotherapy. **f**) Cases treated with some form of adjuvant therapy vs. no or unknown adjuvant therapy. Levels of *p* < 0.050 were defined as significant. See text for further details

Kaplan Meier curves were generated to show the association between different therapeutic variables and OS. In general, the TMZ group was associated with lower HR compared to the pre-TMZ group, nearly reaching significance (HR = 0.795, *p* = 0.067) (Fig. 1a). No significant associations were found in 6 m OS and in 12 m OS between the pre-TMZ and the TMZ groups (*p* = 0.71 and *p* = 0.58, respectively). No significant difference was found between early TMZ and late TMZ cases (Fig. 1b). Both STR and GTR status were significantly associated with favorable prognosis when compared to the status of not having undergone surgery (HR = 0.607, *p* < 0.001 and HR = 0.467, *p* < 0.001; respectively) (Fig. 1c). Finally, treatment with radiotherapy, chemotherapy or any adjuvant therapy were all significantly associated with improved OS (Fig. 1d-f).

**Fig. 2:**
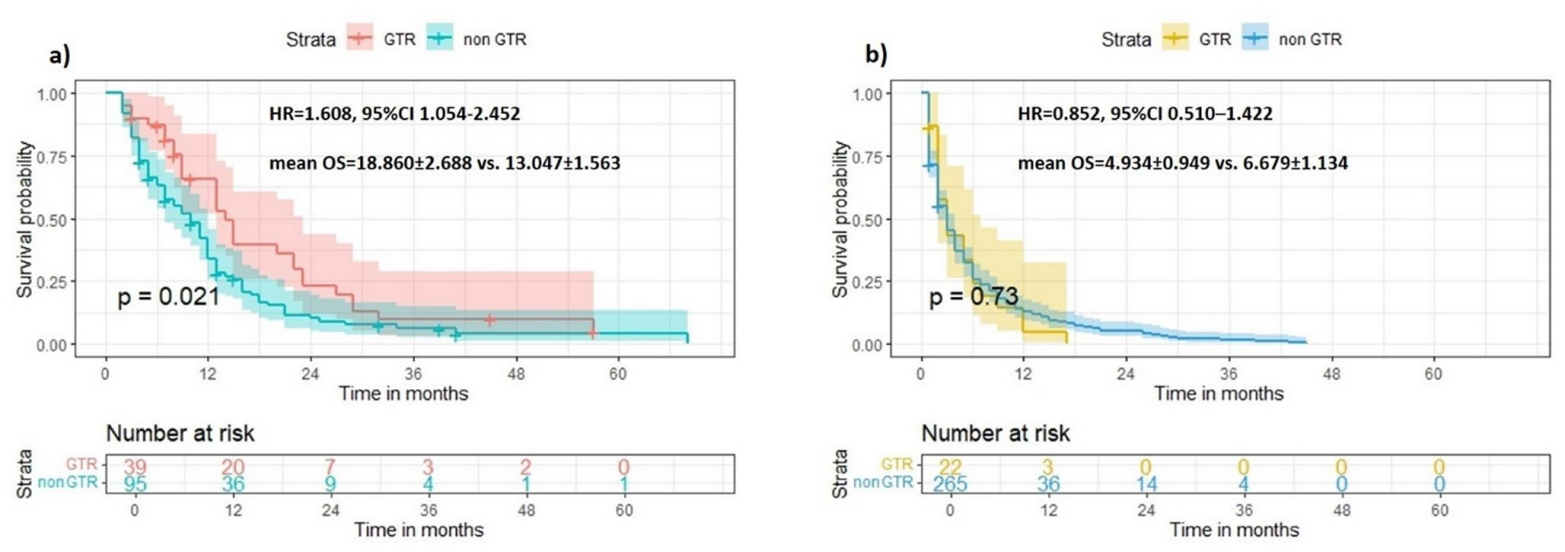
Kaplan-Meier survival curves, comparing GTR to non-GTR status in bGBM patients in two different subpopulations: **a**) patients undergoing surgery followed by combined adjuvant radiotherapy and chemotherapy and **b**) patients undergoing surgery but without combined adjuvant therapy. Levels of *p* < 0.050 were considered significant. See text for further details

In a subgroup analysis of surgical cases, no differences were found between GTR and non-GTR patient subgroups when assessed for OS, 12mOS and 6mOS (HR = 1.316, *p* = 0.095; HR = 1.193, *p* = 0.352 and HR = 1.284, *p* = 0.297). In order to compare these cohorts further, additional analysis was performed, comparing GTR to non-GTR cases based on whether or not adjuvant therapy was given. In the radiotherapy group, GTR (vs. non-GTR) was associated with improved survival, reaching near significance, while no such effect was demonstrated in the no-radiotherapy group (HR = 1.404, 95%CI 0.961–2.050, mean OS = 16.610 ± 2.314 vs. 12.517 ± 1.340, *p* = 0.067 and HR = 0.941, 95%CI 0.499–1.773, mean OS = 3.883 ± 0.692 vs. 5.372 ± 1.068, *p* = 0.831; respectively). Similarly, GTR was significantly associated with improved OS in the group of patients receiving adjuvant chemotherapy, but not in the group not receiving any chemotherapy (HR = 1.587, 95%CI 1.050–2.397, mean OS = 18.505 ± 2.637 vs. 12.837 ± 1.479, *p* = 0.022 and HR = 0.890, 95%CI 0.525–1.509, mean OS = 4.882 ± 0.997 vs. 6.320 ± 1.176, *p* = 0.633; respectively). Finally, GTR to non-GTR cases were compared in bGBM patients who underwent surgical resection and received both radiotherapy and chemotherapy post-operatively, as well as in the subgroup of bGBM patients who did not receive dual adjuvant therapy. This analysis revealed a clear prognostic advantage of GTR in the first group but not in the second (HR = 1.608, 95%CI 1.054–2.452, mean OS = 18.860 ± 2.688 vs. 13.047 ± 1.563, *p* = 0.021 and HR = 0.852, 95%CI 0.510–1.422, mean OS = 4.934 ± 0.949 vs. 6.679 ± 1.134, *p* = 0.73) (Fig. 2).

**Fig. 3:**
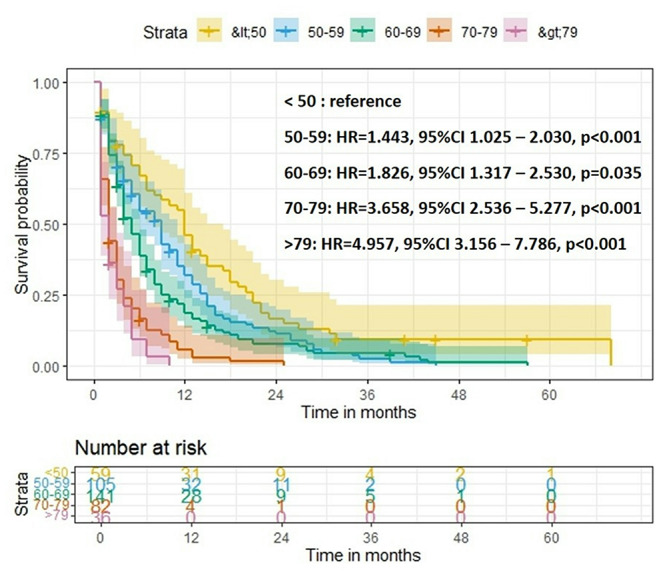
Kaplan-Meier survival curves, stratified by different age groups, showing a significant association between advanced age and worse overall survival. Levels of *p* < 0.050 are considered significant

In the complete cohort of bGBM patients, age was found to be a significant prognostic factor. Figure 3 demonstrates the respective Kaplan Meier curves, stratified by different age groups, showing significant association between older age and worse OS.

However, no significant differences in OS (log rank) were found between GTR and non GTR surgical cases, in any of the age groups investigated.

**Fig. 4:**
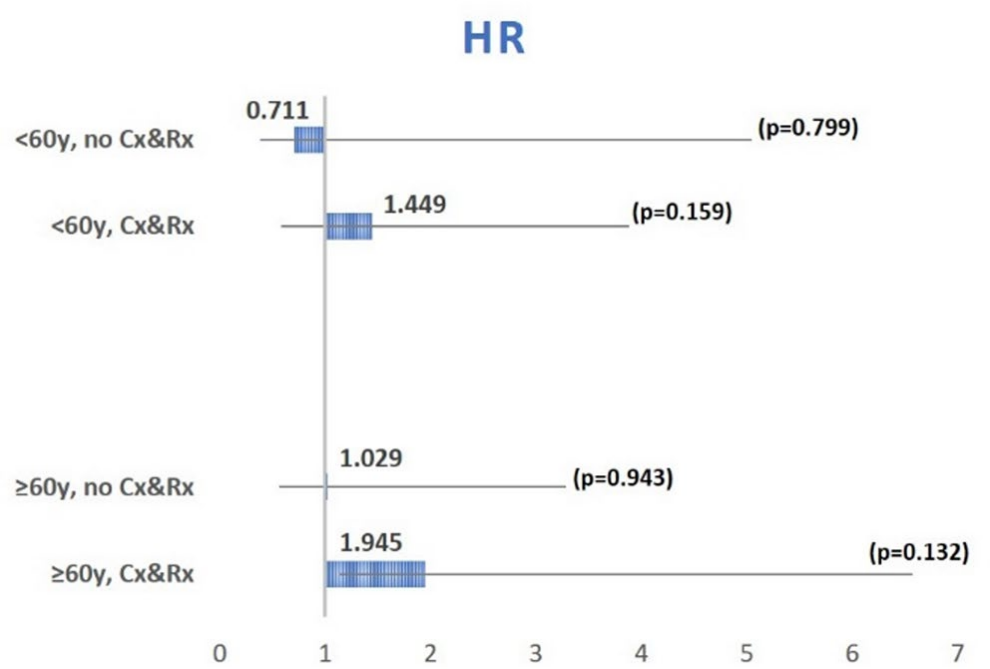
A forest plot graph, schematically illustrating the association between age, extent of resection, adjuvant therapy and overall survival. The hazard ratio (HR) represents the risk of unfavorable OS associated with non-gross total resection. Although no significance was reached for neither subgroup, a trend is noticed toward better OS in cases of combined GTR and systemic therapy. See text for further details. Cx = chemotherapy, Rx = radiotherapy. *p* < 0.050 is significant

When we analyzed subgroups of younger (< 60 years) and older (≥ 60 years) patients, and stratified these by type of surgery (GTR vs. non-GTR) and type of adjuvant therapy, patients who did receive GTR plus any type of adjuvant therapy demonstrated better OS. However, this observation did not reach significance levels in any of the subgroups tested. The association between age, extent of resection, adjuvant therapy and OS is presented in a Forest plot graph (Fig. 4).

## Discussion

In this study, we investigated several clinical management strategies and its impact on survival in patients diagnosed with butterfly gliomas of the brain. To this end, we retrieved a large mixed cohort of adult patients classified as bGBM in the SEER database, spreading over two decades, and have found some significant measures as well as trends in treatment modalities, extent of resection and overall survival.

In general, bGBM cases clinically managed during the era following the establishment of the “Stupp” protocol showed a trend toward better OS. Although this difference only reached near statistical significance (*p* = 0.067), we think this observation carries important clinical significance. Interestingly, the use of chemotherapy was the only therapeutically -related variable that changed significantly before and after the establishment of the Stupp protocol treatment as the standard of care. This may very well have impacted the clear trend toward better OS observed in bGBM patients during the TMZ when compared to those treated pre-TMZ.

Several published reports support the use of chemoradiation in bGBM, even in cases when biopsy only was performed. bGBM patients who undergo biopsy have a short mean OS of 1.3–4.2 months only [12]. A relative recent study reported a mean OS of 7.2 months for other biopsied bGBM cases where improved OS correlated with a high rate of chemoradiation therapy use [9]. Supporting that observation, our data shows that in the TMZ group, both radiotherapy and chemotherapy were significantly associated with improved OS in multivariate cox regression analysis (i.e. irrespective of undergoing surgery or the extent of resection).

When comparisons were made on more homogenous patient cohorts, including only the TMZ era cases, some significant surgically– related variables were found. The rate of actually performed or considered surgery was higher in the late TMZ - vs. early TMZ group, reaching near significance. More importantly, the rate of GTR and the participation rate in any adjuvant therapy was significantly higher in the late TMZ group. However, these differences did not translate in significantly improved prognosis, as both groups shared similar over survival (log rank). One has to keep in mind though, that this may be explained by the relatively small number of patients who underwent GTR in either group (31 vs. 28 in the late and early TMZ subgroups, respectively). Nevertheless, the recorded incidence of GBM as the primary cause of death was significantly higher in the early TMZ group, implying a survival advantage for cases from more recent years. We think that the demonstrated increased rate of surgical consideration for bGBM as well as the associated higher rates of GTR in these cases in recent years are no accident. They reflect the improved pre– and intra-operative technologies alongside with the accumulative data and the clinical implications of the “Human Connectome Project” [13], allowing the safer and more radical resection of these highly challenging cases. Although beyond the scope of this paper, this observational trend is in accordance with the increased number of publications on bGBM in recent years, from 14 “PubMed^®^” publications in the years 1989–2015 to 36 publication in the years 2016–2025.

We then conducted a comparison between GTR and non-GTR subgroups, regardless of the period during which these patients were treated. The GTR subgroup was characterized by younger age at diagnosis and this demographic showed a significantly higher rate of administered postoperative adjuvant therapy which resulted in a significantly decreased rate of GBM as the cause of death.

Importantly, the group of GTR patients who had received any kind of adjuvant therapy (i.e. radiotherapy, chemotherapy or combination of those) showed significantly better OS when compared to the non-GTR patient group. The beneficial impact of GTR on OS was not demonstrated in patients who did not receive adjuvant treatment. This observation is important. We acknowledge that one of the limitations of SEER database is the lack of granular data on patient’s functional status, which has been shown to be a significant prognostic factor in GBM. It is known that patients with higher functional status are more likely to seek and receive adjuvant therapy [14, 15]. By assessing surgically– treated patients who have received adjuvant therapy and by comparing them to those who have not, we have created a more homogenous groups of patients with similar performance status, and therefor may have narrowed the weakness of lacking data on patients’ performance status, overcoming one of SEER’s limitations.

On univariate analysis, reported GTR status was significantly associated with improved survival in both the pre-TMZ and late-TMZ subgroups. Of note, this finding remained significant on multivariate cox regression analysis in the late TMZ subgroup. GTR is increasingly recognized as an important prognostic factor in GBM cases. Notwithstanding the lack of level 1 evidence, maximal safe resection is the main goal of surgery in high-grade gliomas in general [16], with some studies emphasizing the importance of a complete resection for a survival benefit [17].

The classic philosophy regarding of not undertaking any cytoreductive surgical management of bGBMs is based on two assumptions: (1) that all bGBMs are highly aggressive malignancies by nature [18], and (2)the location of bGBMs is critical which makes any attempt at a complete surgical resection a rather risky adventure in a futile situation [3]. However: regarding the first assumption, there is an ongoing debate whether or not all bGBM are intrinsically aggressive. Mistry et al. declared that no study has identified aggressive biological features which are specific to GBMs involving the CC [19]. In addition, a national cancer database study of bGBM [4] showed that MGMT promoter methylation, a known favorable prognostic and predictive factor in GBM, was in fact more prevalent in bGBM patients (47.1% vs. 40.2%). On the other hand, platelet-derived growth factor receptor A (PDGFRA) amplification and missense mutations had higher incidences in ccGBM than in non-ccGBM [20]. Importantly, the HRs of *PDGFRAamp-mut* for PFS and OS were 13.16 (*P* < 0.001) and 16.36 (*P* = 0.003), respectively, suggesting that the poor prognosis associated with bGBM may be related to intrinsic biological and molecular factors [20, 21]. This controversy requires further research. Regarding the second assumption, there is a much greater consensus that bGBM’s historically poor prognosis is secondary to a lack of surgical aggressiveness in the anatomical area of the CC with has resulted in a paucity of data of such patients [2]. Indeed, for long time, bGBM were considered an “inoperable” disease, mostly due to associated severe peri-operative morbidity [22]. Most patients therefore undergo biopsy only to confirm diagnosis and then proceed with palliative medical and radiation treatment. Gradually, however, more and more neurosurgeons have increased their attempts at a more complete extent of resection, showing that it was both safely achievable as well as resulting in improved survival [12, 23]. Tumor resection of localized regions of the CC in itself is unlikely to cause profound loss of neurologic function as evidenced by multiple reports on callosotomy in epilepsy surgery and transcallosal ventricular approaches for resection of tumors [24]. Furthermore, it should be emphasized that bGBM patients who were not selected for surgical resection rarely had a good quality of life for any meaningful length of time. Bifrontal tumor growth and subsequent edema typically caused the patient to become abulic and akinetic shortly after diagnosis, and extensive bifrontal radiation also did not help their executive functional status [23].

In a study on 39 bGBM cases, resection and biopsy were performed in 35.9% and 64.1% of patients, respectively. Resection was found to confer a better prognosis than biopsy (HR 0.37, *p* = 0.009). The minimum EOR needed to observe a survival benefit was found to be 86% (HR 0.054, *p* = 0.030) with a median overall survival of 17.7 months for patients whose EOR > 86% compared to 2.43 months for patients whose EOR ≤ 86% ^2^.

In a recent, most updated, meta-analysis (7 studies, 293 patients), it was shown that surgical resection (compared to biopsy) was associated with improved OS in both the > 80% (HR = 0.27) and < 80% (HR 0.54) EOR subgroups [25]. This meta-analysis also demonstrates the difficulty in achieving solidified conclusions regarding the optimal management of bGBM. The meta-analysis was lacking important data on confounding features such as tumor’s volume, types of second line treatments or radiotherapy features, and tumor-related mutations and gene expression patterns, precluding the authors from conducting a pooled multivariate-adjusted hazard ratio.

Our study encountered similar difficulties. Nevertheless, it is reasonable to state that accumulating data indicate that more frequently reported outcomes from surgeons performing wider resections in bGBM cases are associated with increased rates of patients participating in adjuvant therapies, and some studies have even reported measurably improved OS.

SEER’s database does not contain data on surgical techniques, type of anesthesia and postoperative complications. A literature review has shown that studies reporting a median resection of > 80% presented a similar rate of worsened functional outcomes compared with studies reporting < 80% resection (respectively 33.7% and 35%) [25]. Two studies reported a complete resolution rate of post-operative neurological deficits of 61.5% ^2^ and 66.6% ^4^ and another study stated that when excluding multifocal cases, resection offers a significant improvement in OS in solitary bGBM without a significant increase in postoperative morbidity [5]. Regarding the patient’s functional status as reported by postoperative Karnofsky performance status (KPS), two studies presented no significant difference between biopsy and resection groups [3, 6], which can also be interpreted as microsurgical resection not negatively impacting the patient’s status.

As functional and anatomical knowledge increase and advanced intraoperative technologies become more available (e.g., minimal invasive and endoscopic resection techniques), so will our attempts to achieve GTR become more successful and safer, even in bGBM cases.

In a study comparing the use of multimodal techniques (including neuronavigation, intraoperative MRI, and intraoperative neuromonitoring) with conventional approaches (only guided by neuronavigation) for ccGBM cases, it was demonstrated that multimodally-treated bGBM patients had similar outcomes and survival times to patients with nonbutterfly (= unilateral) ccGBM after resection [26]. Another group has shown that an endoscopic– assisted resection of bGBM yielded a 64% rate of GTR [24]. In a novel study, reporting on awake surgery for bGBM cases, only one patient (1/15, 7%) experienced postoperative abulia following surgery, when using a cingulum-sparing technique. Greater than 90% extent of resection was achieved in 13/15 (87%) of these patients [23].

It should be emphasized that the presented conclusions regarding the prognosis and extent of resection of bGBM do not distinguish frontal from parietal cases. Our study shows that the anatomical location of bGBM (frontal vs. parietal) is not affecting choice and applicability of adjuvant therapy, extent of resection or survival and this is in accordance with previous reports [9].

Our study has found that age is a crucial prognostic factor in bGBM, corroborating the evidence in the literature regarding GBM cases in general [27]. We have also shown that GTR is more likely to be applied in younger patients and that all patients who have received GTR and any type of adjuvant therapy had better OS compared to non-GTR cases. Admittedly, this trend did not reach significance levels for these differences in any of the age subgroups. However, this may be related to the small cohort in each of the groups. No doubt that as the acceptance of bGBM as an “operable” surgical entity will grow, so will our ability to define more precisely which subpopulation of patients and what type of surgery would be most beneficial in terms of neurological outcomes and overall survival. Taking these newer approaches into consideration along with collecting modern molecular data to characterize these rare oncological entities will help us to define the subgroup of patients who will likely benefit from a more aggressive management of this rare and complicated form of GBM.

### Limitations

SEER-based studies carry inherent limitations. The “radiologic” diagnostic of bGBM is based on verbal description of the tumor, without the ability to look at the images. It may be, that not all cases that were radiographically defined as bGBM by our algorithm were indeed true butterfly pathologies. Similarly, the definitions of the achieved extent of resection were based on the primary reports uploaded to the database without the ability to check, revise or calculate the individual tumor volumes pre- and post-operatively to assess volumetric measures. No data is given regarding patient’s functional performance status at time of oncological treatment. Due to the wide acceptance of Stupp protocol as the gold standard for adjuvant therapy in GBM, it is highly reasonable to assume that the majority of patients were treated with the same protocol, and that inclusion criteria (e.g., KPS > 70 as a prerequisite for adjuvant care) would not differ significantly between experienced centers. Nevertheless, no granular data set is available regarding the type of chemotherapy used, the number of cycles applied and the overall duration of treatment. The cause of death should also be read with cautious as it is most likely that in the vast majority of cases, GBM is the cause of death in a cohort of bGBM patients. Finally, there is no data regarding the molecular characterization of the tumors either, which prevented further subgroup analysis of determinants. We hope that these aspects can be addressed in future studies with significantly higher numbers of individuals accrued.

## Conclusions

bGBM cases diagnosed and treated in the TMZ are associated with an increased rate of participation in adjuvant chemotherapy as well as with improved OS, when compared to pre-TMZ cases. Both STR and GTR appear significantly associated with favorable prognosis when compared to the group of patients who did not undergo surgery. The rate of GTR cases has significantly increased in recent years, and is associated with significantly increased rate of post operative adjuvant therapy and with significantly decreased rate of GBM as the reported cause of death. Importantly, GTR patients who have received any kind of adjuvant therapy had significantly better OS when compared to non-GTR patients.

We believe that, based on these observations, bGBM should be treated similar to all other operable GBM cases, using appropriate, advanced surgical techniques in order to achieve the desired maximal safe resection, which then allows patients to move on to adjuvant care.

## Electronic supplementary material

Below is the link to the electronic supplementary material.

Supplementary Material 1

## Data Availability

No datasets were generated or analysed during the current study.
