# Supplementary material for: Butterfly glioblastoma: trends in therapeutic modalities, extent of resection and survival in the temozolomide era. a SEER-based study
Source: Neurosurg Rev. 2025 May 8;48(1):406. doi: 10.1007/s10143-025-03558-2 (PMC12062142; doi:10.1007/s10143-025-03558-2)
Supplement: Supplementary file 1 — Supplementary Material 1 [file 10143_2025_3558_MOESM1_ESM.docx]

**Table 1s.** **Butterfly glioblastoma – differences in extent of resection and oncologic treatments, dependent on anatomical locations: frontal vs. parietal**

|  | **Frontal (n=376)** | **Parietal (n=47)** | **P value*** |
| --- | --- | --- | --- |
| **Surgery**  No  STR  GTR | 244 (52.7)  159 (34.3)  60 (13.0) | 34 (59.6)  14 (24.6)  9 (15.8) | 0.329 |
| **Radiotherapy**  No  Rad w/o Surg  Rad following Surg | 204 (44.0)  148 (31.9)  112 (24.1) | 22 (38.6)  17 (29.8)  18 (31.6) | 0.465 |
| **Chemotherapy**  Performed  No\Unknown | 228 (49.1)  236 (50.9) | 28 (49.1)  29 (50.9) | 1.000 |

GTR=gross total resection; Rad=radiotherapy; STR=subtotal resection; Surg=surgery; w/o=without

* Note: Boldface type indicates p<0.05; Italic type indicated near significance
